# Choice mechanisms for past, temporally extended outcomes

**DOI:** 10.1098/rspb.2014.1766

**Published:** 2015-07-07

**Authors:** Martin D. Vestergaard, Wolfram Schultz

**Affiliations:** Department of Physiology, Development and Neuroscience, University of Cambridge, Cambridge CB2 3DY, UK

**Keywords:** neuroeconomics, experienced utility, decision utility, suboptimal choice

## Abstract

Accurate retrospection is critical in many decision scenarios ranging from investment banking to hedonic psychology. A notoriously difficult case is to integrate previously perceived values over the duration of an experience. Failure in retrospective evaluation leads to suboptimal outcome when previous experiences are under consideration for revisit. A biologically plausible mechanism underlying evaluation of temporally extended outcomes is leaky integration of evidence. The leaky integrator favours positive temporal contrasts, in turn leading to undue emphasis on recency. To investigate choice mechanisms underlying suboptimal outcome based on retrospective evaluation, we used computational and behavioural techniques to model choice between perceived extended outcomes with different temporal profiles. Second-price auctions served to establish the perceived values of virtual coins offered sequentially to humans in a rapid monetary gambling task. Results show that lesser-valued options involving successive growth were systematically preferred to better options with declining temporal profiles. The disadvantageous inclination towards persistent growth was mitigated in some individuals in whom a longer time constant of the leaky integrator resulted in fewer violations of dominance. These results demonstrate how focusing on immediate gains is less beneficial than considering longer perspectives.

## 1. Introduction

Bad decisions happen when people focus on the wrong aspect of the options. An infamous case is when someone, in the pursuit of more, prefers less to more [1]. While good decision-makers choose the option that offers the best outcome or the highest probability of reward, this choice is notoriously difficult for options with outcomes distributed over time [2,3]. In this case, suboptimal outcome can be a result of failure in the summary evaluation of an experience. For example, the decision of whether to revisit a restaurant may be based on a historical evaluation of a previous dinner. The overall value of a dinner is a combination of the values enjoyed from each course [4], but the remembered value of an experience is often dominated by the peak and the final moments [5,6]. However, peak and end values do not represent the overall value of a historical experience when it is later under consideration in the context of choice [7]. Consider a three-course dinner with a mediocre starter, a fine main course and an excellent dessert. If overall value were the sum of the values enjoyed for each course, such a dinner would be equivalent to one with an excellent starter, a fine main course and a mediocre dessert. However, after a disappointing dessert some people feel that the whole experience of the dinner is ruined. This phenomenon has been widely observed in qualitative judgements of both appetitive [8–10] and aversive experiences [11–13]. Most people prefer the ‘happy end’ of an experience, with steadily increasing outcome from start to finish. However, overreliance on recency may lead to violation of dominance: for example, when overall discomfort in medical examinations may be reduced by *adding* an interval of diminishing pain onto the end with the sole purpose of preventing the final experience from being very painful [14]. Thus, in order to assess quantitatively behaviour that violates dominance, we sought to characterize the mechanisms that relate evaluation of perceived, temporally extended outcomes to choices.

Perceived value depends on context [15,16], availability of alternative options [17,18], contrasts to previous or simultaneous rewards [19], and for a temporal sequence it may depend critically on the valence of the most recent temporal contrast [20]. Here, we bring these effects together to develop the hypothesis that decision-makers who favour positive temporal contrasts are prone to choose dominated options. We assessed the retrospective incentive values of temporally extended outcomes using a new monetary gambling task with immediate payoff. Human participants were asked to inspect two alternative streams of virtual coins, whose perceived values were known from a second-price auction. The participants received payout from the chosen stream without experiencing the temporal profile again. Our guiding hypothesis was that incentive value is continuously assessed in relation to temporal contrasts. We assume that perceived value is encoded by a noisy perceptual process and that choice is mediated under noisy discrimination of competing incentives. The development of this hypothesis leads to a stochastic choice model that can predict choice of dominated options for temporally extended outcomes. The critical mechanism is a leaky integrator to characterize the competing incentives. Our behavioural results indicate a distinct overvaluation of positive temporal contrasts over negative contrasts when total value is the same. This preference is so strong that it leads to systematic violation of dominance in a considerable proportion of participants. The strength of this preference is indexed by the time constant of the leaky integrator. Participants with a short time constant are overly impressed by negative contrasts, whereas those with longer time constants are more tolerant and they make better decisions in the long run.

## 2. Methods and material

### (a) Participants

Sixty-one healthy male volunteers participated in the experiments. They were 19–36 years old (avg 26.2, s.d. 3.7), with no history of neurological disorders or psychiatric disease, no self-reported substance abuse or psychoactive medication, and with normal or corrected-to-normal vision. Twenty subjects completed experiment 1 and 41 subjects completed experiment 2. Eleven subjects did experiment 1a and nine subjects did experiment 1b. Eight subjects did experiment 2a and 33 subjects did experiment 2b. One subject in experiment 2b did not seem to participate in the task demands. This subject was excluded from the statistical analyses, but their data are shown in the plots. All subjects completed a pre-experimental evaluation before proceeding to their respective experiment. The participants were recruited to take part in a gambling experiment and were naive to the main purpose of the study. All participants provided written, informed consent. They were paid a fixed fee to participate (£5/hour) plus a variable amount of prize money (£5–15) according to task performance. On completion of the experiment, the subjects received payment in cash.

### (b) Stimuli

In two versions of the experimental task, associations were formed between visual conditioned stimuli (CS) and unconditioned stimuli (US). The CSs consisted of abstract figures composed by arranging randomly squares and triangles of four different colours of equidistant hue, each 50 by 50 pixels. Thus, the CS was 200 by 200 pixels. The USs consisted of sequences of gold and silver coins (stimulus onset asynchrony 350 ms). The coins varied in simulated volume by scaling a reference coin that had a nominal value of 100 pence (£1). Each coin was presented on a background with the same average colour as the coin, so that scaling of the coin did not result in variation in the average colour spectrum of the stimulus. Following each sequence was a visual mask composed by scrambling the image of the reference coin on background. In the pre-experimental evaluation, single coins of varying size were presented for 350 ms.

### (c) Procedures

**Figure 1.**
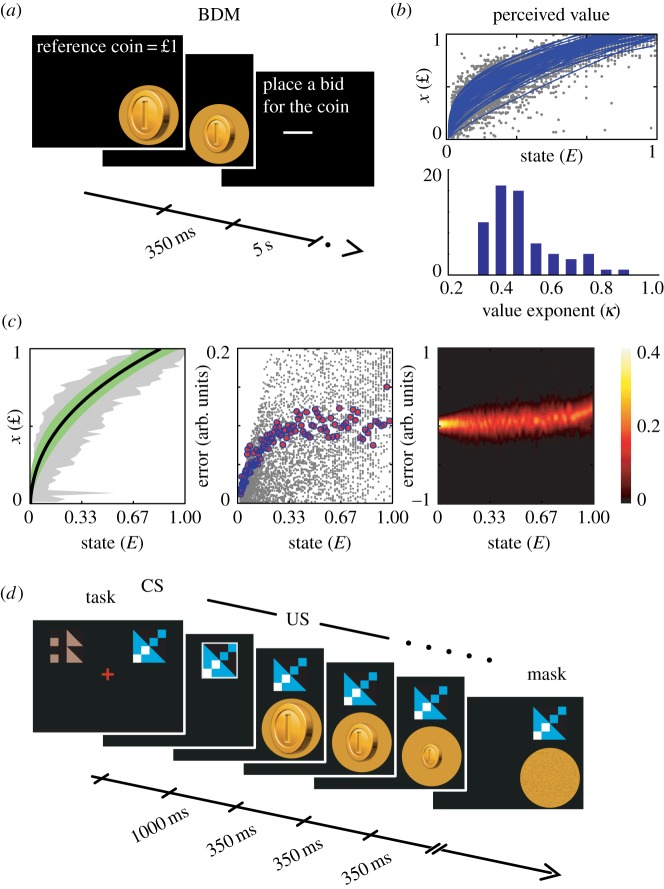
Tasks (*a*,*d*) and behavioural results (*b−c*). (*a*) Evaluations of the virtual coins were obtained by a BDM auction. (*b*) Perceived value estimated from the BDM bids (top) and distribution of value exponents *κ* according to unconstrained fit of the generative value function (bottom). A wide range of value function exponents was observed between participants, with *κ* in the range 0.298–0.846 (mean 0.478, s.d. 0.128). (*c*) Analyses of sampling error shown as the difference between predicted and observed state estimates in arbitrary units (arb. units). The grey area indicates 95% of the variation in state estimates, and the green area indicates the 95% confidence interval of the average value function (left). Subset of positive state errors (grey dots) and standard deviation of the error as a function of state (middle). Error distribution as a function of state (right). (*d*) In the monetary venture games, the competing alternative options are indicated sequentially. A CS precedes each sequence of coins illustrating the instantaneous values at stake (US).

In a pre-experiment, we measured the perceived values of the virtual coins. A basic requirement for deriving perceived value from the physical representation of stimuli is to first measure individual value functions. We used a Becker–DeGroot–Marschak (BDM) auction [21], in which the participants evaluated 120 different coins presented one at a time on a computer screen (figure 1*a*). The participants needed a collection of these coins as gambling tokens in the venture game that followed, and their evaluations served as bids offered on the coins. A £5 budget allowed the participants to obtain a personal endowment of coins according to randomly selected bids. After completing the 120 bids, the participants watched a computer animation of the auction, in which their £5 budget was exchanged into approximately 30 virtual coins. This collection of coins was their initial endowment in the following gambling task. The endowment had an expected value of £10, because the coins were obtained in a second-prize action [22] consistent with the BDM method (for details see the electronic supplementary material, procedures, pre-experimental evaluation).

In experiment 1, we used an exploration task. The participants were shown two CSs and were instructed to choose one freely. Then followed the US, a sequence of silver coins of varying sizes (figure 1*d*). The participants explored the two options in blocks of 20–30 trials within which the association between CS and US remained constant. No feedback was offered. The total value of an option was the sum of the perceived values based on the pre-experimental evaluation, and the profit (loss) gained (incurred) from a block of trials was proportional to how often each option was chosen less the average value. For example, if the total perceived values for two options were £8 and £12 and they were chosen 5 and 15 times, respectively, then the profit would be (5/20) × £8 + (15/20) × £12 – (£8 + £12)/2 = £1. In this way, the value of the endowment could increase or decrease depending on performance (for details see the electronic supplementary material, procedures, experiment 1).

In experiment 2, we used a monetary venture with explicit choice. On each trial, the participants were offered the choice of one of two competing options indicated by two CSs. They were instructed to first inspect one of the options shown by a white arrow. Then followed a sequence of coins of varying sizes (figure 1*d*). Then they would inspect the alternative option. After inspection, the pair of CSs was shown again and the participants indicated which sequence of coins they wanted. It was explained that they should approach the task in the following way: ‘Two pots of money are on offer; first you must inspect the contents of each pot and then choose one or the other.’

Each pair of options consisted of a sequence of gold coins and a subset from that sequence presented either decreasing or increasing. Thus, the options differed quantitatively by the value of the coins omitted from the longer sequence to produce the dominated alternative and qualitatively in the order in which the coins were presented. To vary the salience and make less obvious the underlying temporal profile, two levels of obfuscation noise were used (electronic supplementary material, figure S1 and table S2). In this way, each option was characterized by three features: (i) temporal contrast (positive versus negative), (ii) objective value (dominating versus dominated) and (iii) obfuscation (clear versus opaque). The total value of an option was the sum of the perceived values. One of the sequences was taken from the participant's endowment while the other was on offer from the bank. The chosen pot would go back into the endowment but the sequence was not shown again. In this way, the value of the endowment could either increase or decrease by the difference in total value between the two options, or they could break even, depending on their choice and the respective funding sources for the two options, which they did not know of. The association between CS and US was constant within one trial only (i.e. new CSs and USs were used on every trial). Payment was made by realizing four randomly chosen trials (for details see the electronic supplementary material, procedures, experiment 2).

### (d) Apparatus

The experimental tasks were done in an IAC (Winchester, UK) double-walled, sound-attenuated test booth, and the stimuli were presented on a 17-inch Dell LCD screen with a refresh rate of 60 Hz. The participants responded to the task demands using an ordinary keyboard. Experiments, stimuli, statistical analyses and modelling were done in MATLAB (Release 2011b) v. 7.13.0.564 using the Image Processing Toolbox v. 7.3, Optimization Toolbox v. 6.1 and Signal Processing Toolbox v. 6.16.

## 3. Results

### (a) Stochastic mechanisms underlying suboptimal choice

The relationship between a stimulus and its perceived value is given by a value function. A central requirement is that the value function is continuous, monotonous and concave [23]. Moreover, it is widely accepted that there is a power relationship between a stimulus's magnitude and the signal encoding its perceived intensity for many sensory modalities [24–26]. These considerations make it physiologically plausible that the perceived value *x* derived from a reward stimulus is given by a compressive power function,
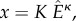
where 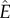 is the observer's estimate of reward magnitude and *K* is a scaling coefficient. The critical parameter is *κ*, which describes how compressive is the translation from physical state to perceived value. The pre-experimental evaluations showed that *κ* was between 0.3 and 0.8, confirming that the value function for the virtual coins was increasing and concave (figure 1*b*). The parameters of individual value functions were estimated using unconstrained 2-norm minimization of the state error in the pre-experimental evaluation. Using these data, we analysed the underlying noise in state estimate (figure 1*c*). This analysis indicated that state is sampled under noise with constant bounds except from a floor effect for small coins, where the error has an upper bound of the size of the coin. In the following tasks, all participants experienced the same amount of identical coins, but their monetary values were calculated according to individual value functions.

An episode of temporally extended outcomes consists of a sequence of stimuli *E*(*t*) experienced as a sequence of perceived values *x*(*t*). Optimal incentive would be simply the integration of *x* over *t*, that is the sum of the perceived values over the duration of the episode. However, the bounded dynamic range of neural encoding of stimuli makes this operation computationally intractable for episodes of unknown duration. Moreover, under uncertainty people often rely on simple judgmental operations according to the so-called availability heuristic [27]. In keeping with these considerations, we propose that people continuously track the incentive value *y*(*t*) of perceived, temporally extended outcomes in relation to historical incentive. The change in incentive value dy/d*t* is the perceived value *x*(*t*) experienced in relation to the previously accumulated incentive
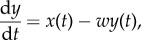
where *w* is a weight parameter for characterizing the immediacy of the running contrast on the marginal incentive. The critical feature of this representation is that current incentive continuously serves a reference point for the evaluation. Accumulation of evidence in terms of incentive value therefore occurs as leaky integration of the perceived values by means of an exponential filter with decay parameter *τ* = 1/*w* (the complete derivation is given in the electronic supplementary material, decision model, which also refers to alternative value functions and choice mechanisms).

The leaky integrator instantiates a stable Ornstein–Uhlenbeck process, which has also been proposed as a model for sequential sampling in perceptual [28,29] and multi-attribute [30–32] decisions. It can account for spike sequences of cortical neurons [33] and order effects [34] in perceptual choice. For temporally extended outcomes, the perceived and incentive values are available to the observer throughout the episodes. In subsequent binary choice, the two competing incentives are compared in a noisy decision process by logistic discrimination of the accumulated evidence. This choice model provides a sufficient framework for analysing choice of previously experienced temporally extended outcomes with the potential to account for violation of dominance.

**Figure 2.**
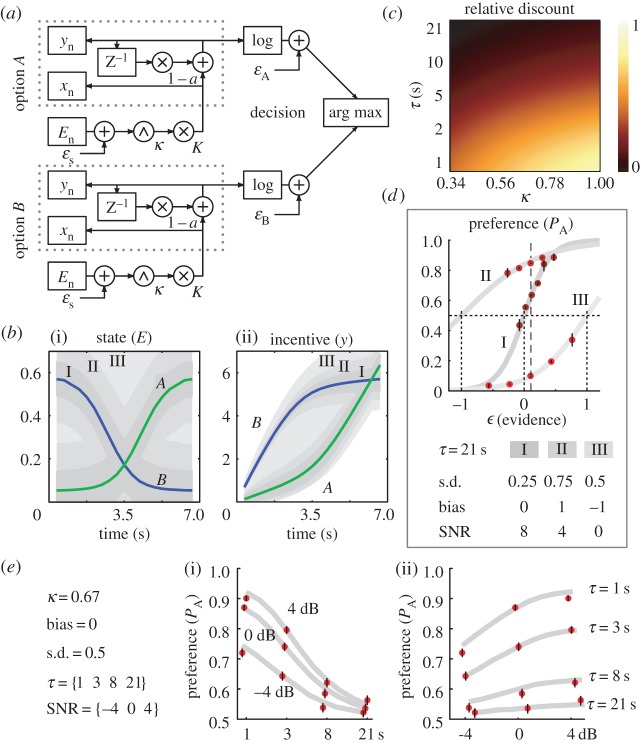
Stochastic value coding by suboptimal integration predicts choice of dominated options. Simulations (*n* = 15 000) in (*b*,*d*,*e*). (*a*) The state (*E*_n_) of each option is sequentially observed under sampling noise (*ɛ*_S_). The value function encodes state estimate (*E*_n_ + *ɛ*_S_) as perceived value (*x*_n_). Incentive value (*y*_n_) is accumulated by suboptimal integration of the perceived values with decay parameter *τ* =−1/log(1−*a*). Choice is mediated under noisy discrimination of the evidence *ε*_A_ = log (y_A_/*y*_B_). The decision noise components 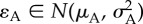 and 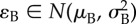 predict decision bias (*β*) and sensitivity (*B*) of the agent, *β* = *μ*_A_ − *μ*_B_ and 
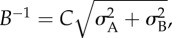
 where *C* is a scaling constant for aligning the logistic and normal distributions. Thus, there are two free parameters, *β* and *B*, derived from the decision noises. (*b*) Decreasing and increasing temporal profiles characterizing two options with identical contents (i) but with differential cumulative incentive for *τ* = 21*s* (ii), expected value of evidence, EV(*ε*_A_) = 0.08. The grey areas show two-sigma bounds of the sampling noise for signal-to-noise ratios, SNR ∈ {0, 4, 8 dB}. (*c*) Difference in expected incentive relative to linear integration for a range of value function exponents (*κ*) and integration decays (*τ*). (*d*) Psychometric functions (grey) and simulations relating evidence for the increasing profile to choice probability for three parametric implementations (I, II, III). EV(*ε*_A_) and indifference points for evidence are indicated by dashed and dotted vertical lines, respectively. Average preference (±s.e.m.) shown in five bins equally distributed around EV(*ε*_A_). (*e*) The effect of SNR and integration decay for a fixed value function (*κ* = 0.67), predictions (grey lines, based on EV(*ε*_A_) without decision noise) and simulated choice data (average ±s.e.m., incl. decision noise). The grey curves approach the asymptotes determined by the leaky integrator for low *τ* (i) and for high SNR (ii).

We consider the mechanism in the discrete domain (figure 2*a*). The contents of each option are sequentially observed under sampling noise, and a value function encodes the observed stimuli as perceived values (*x*_n_). Meanwhile, the incentive values (*y*_n_) are accumulated suboptimally by leaky integration of the perceived values, and subsequent choice is mediated under noisy discrimination of the final evidence *ε*_A_ = log (*y*_A_/*y*_B_).

To illustrate the mechanism, we first summarize the results from a simulation. We then report the results from a series of human choice experiments using the new gambling task with immediate monetary payoff. The parameters of the choice model are fitted to the human choice data in order to characterize individual evaluation strategies. The simulation confirms the theoretical foundation, while the human data provide empirical justification. The results show that this mechanism can explain suboptimal choices for temporally extended outcomes.

### (b) A computational model of choice behaviour

We simulated choice of temporally extended outcome using competing options with identical contents. Thus, the total values in every pair of options were the same so optimal discrimination would lead to indifference. The options differed only in whether the contents were arranged along decreasing or increasing temporal profiles (figure 2*b*(i)). Systematic preference for one of the options would therefore not be justifiable from the total perceived values but from a difference in the incentive values. Neural signal-to-noise ratio (SNR) was simulated by sampling noise, and sensitivity to variation in evidence was simulated by decision noise (for details see the electronic supplementary material, simulations).

The leaky integrator discounts historical values (*x*) in the running calculation of the incentive (*y*) for each option. This mechanism leads to emphasis on recently perceived values by favouring the option with positive temporal contrasts (figure 2*b*(ii)). The difference in retrospective discount between the two alternatives determines the difference in incentive value. The less concave is the value function, and the shorter is the decay, the more is the relative difference in incentive value (figure 2*c*; electronic supplementary material, figure S2). Sampling noise causes nonlinear distortion of the perceived and incentive values (figure 2*b*), but it does not affect the expected value of the evidence (figure 2*d*). Thus, for options of equal contents varying only in temporal profile, sampling noise simply reduces the effect of the decay parameter *τ* (figure 2*e*). The specification of decision noise determines the steepness and offset of the psychometric function in a predictable way, characterizing the sensitivity and bias of the chooser (figure 2*d*). Note that optimal accumulation of evidence is given for *τ* → ∞.

The simulation confirms the expected mechanism of the main parameters. The value function defines the translation from physical state to perceived value, which in turn affects the effect size for the other parameters. Sampling noise reduces the impact of the expected value of evidence regardless of whether it reflects a difference in perceived or incentive values, while decision bias and leaky accumulation of evidence can generate differential preference for certain temporal profiles.

### (c) Human choice behaviour

To examine the extent to which preference for positive contrasts leads to suboptimal outcome in humans, we recruited participants to partake in a gambling experiment. The stimuli were three-dimensional renditions of gold and silver coins of different sizes presented on a computer screen. To ensure that bigger coins were always perceived as more valuable, the value function was continuous, increasing and non-satiated. The perceived values of the virtual coins were known from the pre-experimental BDM evaluation (figure 1*a*). The coins were used as gambling tokens in a rapid venture game, in which one of two competing monetary options was always weakly dominated.

We investigated the competing roles of temporal contrasts and duration for sequences of coins that varied in size along a temporal profile. On each trial, two pots of coins were on offer, and the participants would indicate their preferred sequence. The options contained varying amounts of coins (up to 19) presented at approximately three coins per second (stimulus onset asynchrony 350 ms). This pace was too fast for estimating and summing up the perceived value of every single coin; consequently, the participants were instructed to form overall impressions of the total value of the competing options. We used two different versions of this game, with implicit and explicit measures of preference, respectively. The first version assessed effects of shape and coin position in a variety of temporal profiles.

**Figure 3.**
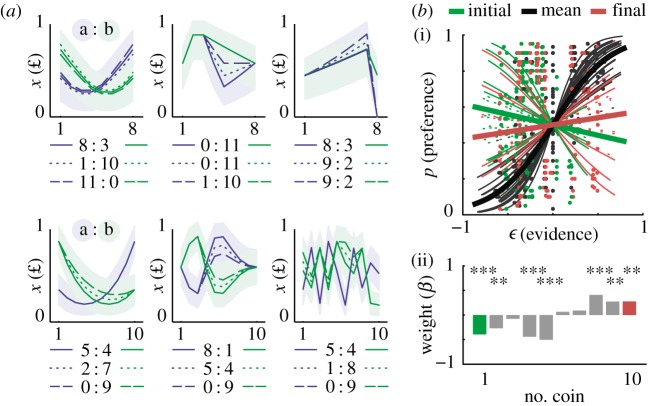
The effects of shape and coin position for profiles of temporally extended outcome. (*a*) Competing profiles with 7–10 coins varied slightly as indicated by line type (in some cases, dotted and dashed lines are behind the solid lines); omitted coins are shown as £0. Overall preference ratios (numbers of subjects) are indicated below each set. (*b*) Multiple regression for evidence derived from initial, mean and final values; (i) individual subject data and group averages are shown and (ii) fixed effects analysis of coin position. To enable the latter, all temporal profiles were upsampled to 10. Regressors were orthogonalized to evidence according to mean value. This analysis conserves any correlation between the proxies and it removes effects of mean value. *p < 0.05, **p < 0.01, ***p < 0.001.

In the first version of the game, 20 participants completed the exploration task (experiment 1). The participants explored pairs of coin sequences freely in blocks of 20–30 trials without feedback, and preference was inferred implicitly by choice frequency in a block. We observed strong preference for increasing profiles and for some dominated options characterized by the omission of a small extra coin at the end (figure 3*a*). Multiple logistic regression assessed if observed choices were determined by evidence derived from simple proxies for total value, such as the initial, average or final values. A significant role was observed for the single initial (*b* = −0.43, *p* = 0.0000053) and final (*b* = 0.30, *p* = 0.0018) values, while strong predictive leverage on choice was observed for average value (*b* = 2.79, *p* = 1.4 × 10^−149^; figure 3*b*(i)). These results indicate that the participants were sensitive to the chronologic configuration of the outcome. The results are consistent with the hypothesis that incentive value is related to the succession of temporal contrasts; starting low and finishing on a high is preferred over the reverse (figure 3*b*(ii)). The following experiment assessed the extent to which this preference competes with sequence duration.

In the second version of the game, 41 participants completed the monetary venture task with explicit choice (experiment 2). As before, the participants were offered two sequences of coins of varying sizes and indicated which sequence they would prefer. But this time, they would first inspect the two options with no commitment, and then afterwards indicate explicitly which one they preferred. The chosen sequences were not shown again. Options presented coins in sequences with mainly positive or mainly negative temporal contrasts like the ones we used in the simulations (figure 2*b*), either dominating or dominated by the alternative option. Some options had exactly the same contents and differed only in being presented along different temporal profiles. While most options varied in duration, they all had the same average objective value. In this way, we examined choice behaviour for monetary options with two main attributes: temporal contrasts (positive or negative) and sequence duration (dominated or dominating contents). Importantly, these attributes could either be incentivizing the same option or they could conflict (e.g. a long sequence of negative contrasts).

We first examined choice performance for attributes that were in conflict. We offered long sequences with decreasing temporal profile against short sequences with increasing profile (electronic supplementary material, figure S1*a*–*d*). In this case, preference for the dominating option was not significantly different from chance performance (avg 0.57, *t*_7_ = 1.87, *p* = 0.10). By contrast, there was strong preference for dominating flat sequences without temporal contrast (electronic supplementary material, figure S1*g*; avg 0.79, *t*_7_ = 4.3, *p* = 0.0034), and there was an equally strong preference for positive contrasts for sequences with no difference in duration (electronic supplementary material, figure S1*h*; avg 0.78, *t*_7_ = 5.1, *p* = 0.0014). In this special case, in which the attributes were in conflict, preference for positive contrasts was effectively modelled as either decision bias towards the option with positive contrasts or as leaky integration of evidence (electronic supplementary material, table S3).

**Figure 4.**
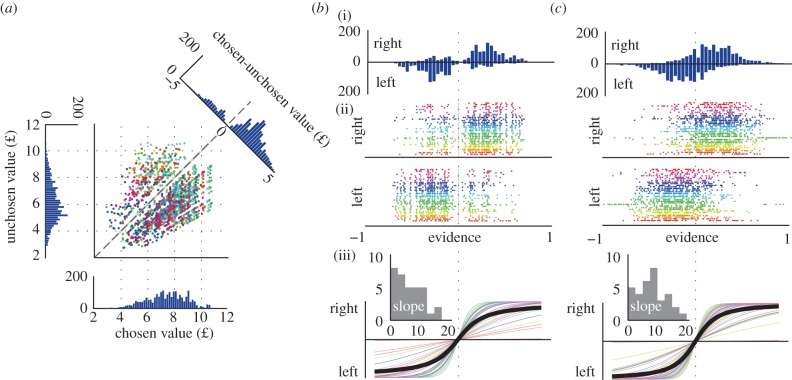
Gains and losses, and the underlying observed and modelled evidence (each colour shows data from one participant). (*a*) The distributions of chosen and unchosen values and the bimodal difference distribution. (*b*) Distribution of evidence according to the null model across (i) all participants, (ii) individual data, (iii) logistic discrimination of evidence (individual psychometrics in colour and group average in thick black) and (inset) distribution of slope estimates. (*c*) As (*b*) but for evidence according to incentive value accumulated by leaky integration of perceived value.

To examine which mechanism (biased decisions or leaky integration of evidence) was the more likely determinant of dominated choice, we presented the option attributes in a balanced manner. A coin sequence could be increasing or decreasing, and it could be long or short in all four combinations (electronic supplementary material, figure S1*c*–*f*). In this case, the average choice frequency for the dominated option was 0.21 (s.d. 0.15). Choice of the dominated option was more frequent when the attributes were in conflict (avg 0.29, s.d. 0.23) than when in concord (avg 0.13, s.d. 0.13) (*F*_1,31_ = 19.8, *p* = 0.0001, 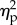 = 0.39; electronic supplementary material, figure S3). The average RT in the experimental conditions was 1.11 s (s.d. 0.28 s), with no significant differences between conditions. The average total value of the chosen options was £7.23 (s.d. £1.07), whereas for unchosen options it was £6.30 (s.d. £0.98; figure 4*a*). When the better option was chosen, an average profit of £2.10 (s.d. £1.00) was gained, whereas the average loss for bad choices was £1.77 (s.d. £0.95). After the experiment, most participants reported a tendency to have classified the coins roughly in size (e.g. small or large; see the electronic supplementary material, response strategies). They typically revealed to have looked for the options with most large coins while trying to ignore the apparent temporal profile. Still, substantial direct loss was incurred in this situation, in which profit maximization depended entirely on sequence duration, with the temporal contrasts merely providing supporting or conflicting incentive.

### (d) A model of human choice behaviour in rapid monetary judgement

**Figure 5.**
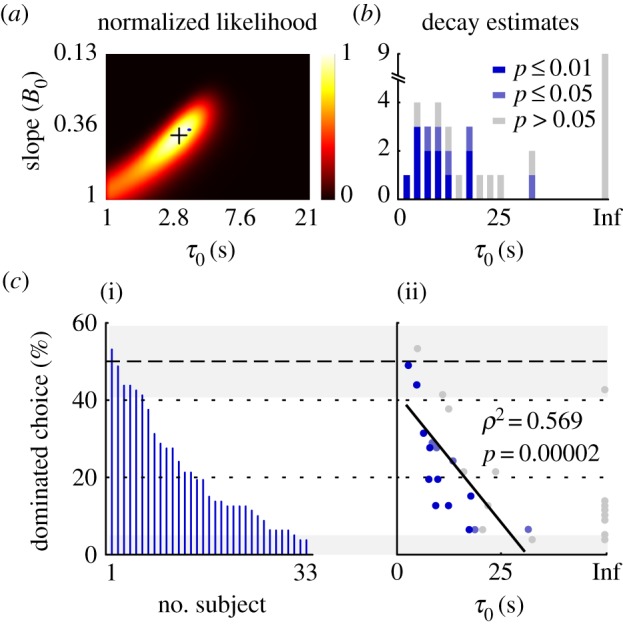
Leaky accumulation of evidence causes preference for dominated choice options. (*a*) Normalized likelihood surface for decay (*τ*_0_) and slope (*B*_0_) in one subject. The orthogonal bars at the peak mark the Hessian; also shown is the posterior mean by the blue dot. (*b*) Distribution of the decay parameter for all subjects. (*c*) The scale of dominated choices ordered by (i) propensity and (ii) relationship between dominated choice and decay estimates. Cases in which *τ*_0_ = Inf were excluded in the calculation of the correlation. Including these cases, Spearman rank correlation was *r*^2^ = 0.551 (*p* = 1.04 × 10^−6^).

The generative model was fitted to the choice data to investigate the involvement of decision biases and evidence decay. The perceived values of each sequence were calculated according to error-free individual value functions estimated from the pre-experimental auction. Evidence was derived according to each model, and parameter estimates were obtained by maximizing the likelihood of the choice data given the model's evidence (figure 5*a*). We examined effects of bias for the option first inspected (*β*_1_), of bias for positive contrasts (*β*_+_) and of leaky integration decay (*τ*). We used six separate models implementing decay and biases in a 2 × 3 factorial way (decay/no decay (*τ*) × no bias/primacy (*β*_1_)/positive contrasts (*β*_+_)). All models include as a free parameter the inverse temperature (*B*) of the stochastic decision process, so in total the models had 1–3 free parameters (*τ*, *β*_,_ *B*). We fitted all six models and report the number of participants (*n*) in whom the different models provided the best fit according to the Akaike information criterion (electronic supplementary material, figure S5). The null model (no decay, no bias) was best in five participants. The most successful model (*n* = 13) was the bias-free leaky integrator that captures evidence suboptimally by favouring a succession of positive contrasts. Second most successful (*n* = 8) was the model with bias for positive contrasts. Eight participants accumulated evidence optimally, leading to *τ* → ∞. The average decay in the remainder was 12.2 s (s.d. 8.5 s; figure 5*b*). When the models are compared at the group level, the best model was the bias-free leaky integrator (mean group AIC difference of five compared with the null model; electronic supplementary material, table S4). Leaky integration of the perceived values leads to better sensitivity to variation in evidence compared with non-leaky integration (figure 4*b*,*c*). These results depend in no way on qualitative judgement of the choices; they are simply the best statistics to describe the observed behaviour. Even so, there was a strong negative correlation between the decay factor and dominated choice score (*ρ*^2^ = 0.57, *p* = 0.00002; figure 5*c*; electronic supplementary material, figure S4). Dominated choice for participants who accumulated evidence suboptimally was 0.24 compared with 0.099 (*t*_30_ = 2.6, *p* = 0.014) for those in whom *τ* → ∞. While there was no significant correlation between value function exponent and behaviour, model fitness and parameter estimates depended critically on the individual value functions (electronic supplementary material, figure S6). Thus, a decay factor *τ* that discounts the perceived values is the most probable mechanism to underlie the systematic choice of dominated options in these experiments.

## 4. Discussion

This study shows that preference for dominated monetary options of temporally extended outcomes occurs as a result of suboptimal accumulation of evidence. When people fail to appreciate the extended value of an experienced sequence of outcomes they may later choose a dominated option. Systematic bad decisions in our rapid gambling task are therefore the aftermath of a misrepresentation of historical values leading to overvaluation of recently perceived values. While order effects in decision-making [35,36] and evidence accumulation [37,38] classically include both recency and primacy, we observed no role for primacy-based evidence weighting in retrospective evaluation. Violation of dominance has also been reported in predicted preferences for future rewards [39–44]. However, since retrospective valuations differ distinctly from hypothetical future outcomes, we have restricted our investigation to experienced past outcomes and focused on incentive-compatible representations of perceived value. Although previous studies have suggested that humans and other animals are generally optimal decision-makers [45,46], our data demonstrate that many people systematically prefer lesser-valued options involving successive growth over better options with decreasing temporal profiles. Our choice model shows that the critical underlying mechanism is leaky integration of the perceived values. It favours recency through robust preference for successive positive contrasts and aversion to negative contrasts. This disadvantageous inclination towards persistent growth was effectively characterized in two ways: either directly as the proportion of dominated choices (figure 5*c*(i)) and the associated premium incurred (figure 4*a*), or indirectly as decision bias and short time constants in the accumulation of evidence predicting choice behaviour. Together, these results provide a controlled account for violation of dominance in perceived, temporally extended outcomes and demonstrate in a formal manner how focusing on immediate gain is less beneficial than considering longer perspectives.

### (a) Mechanisms underlying dominated choice

Sensory noise interferes with the initial encoding of stimulus magnitude prior to evaluation. Likewise, leaky integration of value is a mechanism at the level of evidence accumulation. By contrast, decision bias is related to interpretation of evidence. We discuss these mechanisms separately.

The first mechanism is inaccurate sampling of the state that represents the options. Sensory noise causes stochastic misrepresentation of the perceived values. Our simulations show that the distortion of evidence caused by sensory noise is symmetric around the expected value. Sensory noise may lead decision-makers astray by incentivizing an option of lesser value, but just as often it may boost evidence for the option that was better anyway. Thus, sensory noise does not generate systematic preference reversals or impede rational decision-making for temporally extended outcomes in any systematic way.

The second mechanism to consider is decision bias (i.e. the expected value of decision noise). In this case, the internal representation of evidence for the options feeds a decision process that penalizes evidence for a particular option, for example one with a bad end. Our behavioural data show that decision bias is sometimes a plausible mechanism to model violation of dominance. In those cases, the decision-maker may even have access to the fact that evidence is in favour of the unpreferred option, while all the same they commit to the alternative in a biased decision. Consider the inner voice of the biased decision-maker arguing: ‘Option B seemed better, but I really like option A, which ended so well.’ The biased decision-maker allows option B to seem better to a certain extent and still choose option A with the happy end. Thus, decision bias is a plausible mechanism in some choosers, perhaps acting according to the availability heuristic [27].

The third mechanism we examined was suboptimal accumulation of evidence. Leaky integration of value occurs as a result of contrast-guided evaluation, and our simulations show that leaky integration of perceived values will lead to evidence in favour of positive contrasts (figure 2*b*). Moreover, the effect of a sequence's duration on its incentive value is diminished due to the leak. Thus, it is a sufficient mechanism for explaining preference for dominated options with an increasing temporal profile. This mechanism represents an extension of the ‘end rule’, according to which the final value in a sequence assumes an overriding role over historical values [47]. To illustrate the difference from biased decisions, the inner voice now argues: ‘Option A seemed better overall although option B was longer.’ The unbiased decision-maker neglects duration [9], because he is so favourably impressed by the succession of positive contrasts. The human choice data were consistent with this mechanism.

Leaky integration of perceived values and decision biases are independent mechanisms. While the former implements the accumulation of evidence, decision bias is the amount of evidence to the contrary that the decision-maker allows when committing to a choice. Therefore, the two mechanisms may coexist, as also seen in three participants who exhibited leaky integration of evidence along with decision bias towards the option first inspected (electronic supplementary material, figure S5). Overall, violation of dominance was better accounted for by a generative process that integrates evidence suboptimally via leaky integration of the experienced values than by a generative process that integrates evidence optimally only to feed it into a biased decision process. Thus, suboptimal choice for temporally extended outcomes is more probably the result of leaky accumulation of evidence than of decision biases or sensory noise.

Our results are comparable with the dichotomy of ‘experienced utility’ and ‘decision utility’ [3]. Classically, experienced utility is thought to be the hedonic impact of the constituents of a temporally extended outcome. Kahneman and Tversky observed that experienced utility was not a good predictor of retrospective preferences, which indicated an option's decision utility. Decision utility is largely thought to be the internal representation of remembered utility in the context of choice. We have assumed that perceived value is represented internally in the absence of choice (e.g. during the BDM evaluation), just like experienced utility [48]. Although BDM evaluations of virtual coins do not represent hedonic impact, they signify the incentive-compatible values that decision-makers should integrate in order to optimize their return. Thus, incentive value as defined above is the critical input to the decision process in much the same way as decision utility is thought to have informed the participants in Kahneman and Tversky's famous experiments.

Although contrast-guided evaluation can lead to suboptimal behaviour in some experimental settings, it is conceivable that sensitivity to temporal contrasts has an evolutionary basis. The idea that a positive contrast signals something even better coming up is ecologically plausible. Thus, temporal contrasts may be honest indicators of the prospect for slowly varying events [49]. Positive and negative contrasts can therefore serve as reliable signals for optimizing fitness. According to this notion, there is survival value in the repulsion to negative contrasts. Such a mechanism would be critically supported by the strong tendency of animals to approach stimuli associated with rewards and to withdraw from stimuli associated with danger [50]. Therefore, contrast-guided evaluation may be an ecologically viable strategy for slowly varying events. However for hasty monetary decisions such as in our gambling task, an inclination in favour of persistent growth is disadvantageous. The ensuing behaviour is characterized by a ‘banker's fallacy’, which is the propensity to focus disproportionately on immediate growth in economic decisions when tolerance to temporary decline would result in more profitable transactions.

## Supplementary Material

ESM

## Data accessibility

Data supporting this article may be requested by contacting M.D.V. (mdv23@cam.ac.uk).
