# Supplementary material for: Choice mechanisms for past, temporally extended outcomes
Source: Proc Biol Sci. 2015 Jul 7;282(1810):20141766. doi: 10.1098/rspb.2014.1766 (PMC4590462; doi:10.1098/rspb.2014.1766)
Supplement: ESM [file rspb20141766supp1.pdf]

**Choice mechanisms for past, temporally extended outcomes**

*Martin D Vestergaard, Wolfram Schultz*

**Supplementary material**

## Decision model

*Perceived value*

The perceived value ($x$) of a reward stimulus scaled to magnitude $m$ is given by Stevens' Power Law [1]:

$$x = A\, m^{\alpha}$$

where $A$ and $\alpha$ are individual parameters of the observer. For $A > 0$ and $0 < \alpha < 1$, the power value function is increasing and concave. It is a case of the isoelastic value function:

$$u(m) = \frac{m^{1-\eta}}{1-\eta} \text{ for } \eta \neq 1$$

with elasticity coefficient $1 - \eta$ and relative risk-aversion coefficient $\eta$ according to the Arrow-Pratt criterion. Our value function is restricted to the case $\eta < 1$; that is we do not consider cases where $A$ and $\alpha$ are negative. As an alternative to isoelastic value functions, the negative exponential value function is sometimes used to analyse choice behaviour under uncertainty. It has the form, $u(m) = 1 - \exp(-\eta m)$ with constant absolute risk-aversion $\eta$, which means that risk attitude is independent of current wealth. The negative exponential value function is satiated for high reward magnitude, which means that variation in reward magnitude might not lead to variation in perceived value, and because of that we favour the isoelastic value function. Satiated value functions are not compatible with the sort of preference patters we would like explain.

The state variable ($E$) that relates our stimuli to perceived value is volumetric magnitude. The stimuli are sequences of virtual gold and silver coins, each coin of maximum value £1 (100 pence) and each coin scaled in size by a factor $m$. The sequence of perceived states is:

$$\hat{E}(t) = 100\, m^3(t) + \varepsilon_s$$

where $\varepsilon_s$ is sensory noise in the observer's sampling of the states. It is assumed that the error lies in sampling of the state and not in the implementation of the value function. The value function relating a sequence of virtual coins of scales $m(t)$ to perceived value is:

$$x(t) = K\, E^{\kappa}(t)$$

where $\kappa = \alpha/3$, $K = A/(100^{\kappa})$.

*Incentive value*

The differential equation describing incentive value belongs to a class used in a wide range of problems from electrical engineering to membrane physiology [2]. In this case, we propose that economic agents continuously track the incentive value, $y(t)$ of a sequence of temporally extended outcomes in relation to historical incentive. The change in incentive value, $dy/dt$ is the perceived value, $x(t)$ experienced in relation to the previously accumulated incentive:

$$\frac{dy}{dt} = x(t) - wy(t)$$

where $w$ is a weight parameter for characterizing the immediacy of the running contrast on the marginal incentive. The homogeneous solution is:

$$y(t) = e^{-wt} \int x(t) e^{wt} \, dt$$

The retrospective incentive value of an episode of duration $T$ is thus:

$$y(T) = \int_0^T x(t) e^{\frac{t-T}{\tau}} \, dt$$

where $\tau = 1/w$. The decision variable, $\rho_\mathrm{A}$ for two options (A and B in Fig. 2a) is the incentive value ratio:

$$\rho_\mathrm{A} = \frac{y_\mathrm{A}}{y_\mathrm{B}}$$

In order to compare retrospectively two options evaluated sequentially in the past, the agent discriminates the evidence (in favour of option A):

$$\epsilon_\mathrm{A} = -\log\left(\frac{1}{\rho_\mathrm{A}}\right)$$

Preference for option A, $P_\mathrm{A}$ follows from logistic discrimination of the evidence:

$$\log\left(\frac{P_\mathrm{A}}{1 - P_\mathrm{A}}\right) = B(\epsilon_\mathrm{A} + \beta_\mathrm{A})$$

where $B$ is the inverse temperature and $\beta_\mathrm{A}$ is decision bias in favour of option A. Logistic discrimination is functionally equivalent to Softmax activation for binary choices. The parameters $B$ and $\beta$ describe the sensitivity and predisposition of the decision maker to gauge the evidence for and against the two choice options.

Accumulation of evidence in terms of incentive value, $y(t)$ occurs as integration of perceived value, $x(t)$ by means of an exponential filter, $h(t)$ with decay parameter $\tau$. The Laplace

transform filter is $\mathcal{L}[h(t)] = [z + 1/\tau]^{-1}$ and the filter's impulse response is the exponential decay function:

$$h(t) = e^{-\frac{t}{\tau}}$$

which specifies the retrospective period weights to the sequence of perceived values in the computation of total incentive. A schematic circuit of this mechanism is illustrated in the dotted boxes in Fig. 2a. The state variables *E* pertaining to two options of temporally extended outcome are expressed in terms of perceived value, *x* and incentive value *y*. The perceived and incentive values are available to the agent during the episodes at the end of which the competing incentives are discriminated under noisy comparison. The decision noise components $\varepsilon_{\mathrm{A}} \in N(\mu_{\mathrm{A}}, \sigma_{\mathrm{A}}^2)$ and $\varepsilon_{\mathrm{B}} \in N(\mu_{\mathrm{B}}, \sigma_{\mathrm{B}}^2)$ specify the bias ($\beta$) and sensitivity ($B$) of the agent, $\beta_{\mathrm{A}} = \mu_{\mathrm{A}} - \mu_{\mathrm{B}}$ and $B^{-1} = C\sqrt{\sigma_{\mathrm{A}}^2 + \sigma_{\mathrm{B}}^2}$, where $C$ is a scaling constant for aligning the logistic and normal distributions. The cumulative logistic distribution with variance $\sigma^2 = 1$ is given for $C = \sqrt{3}/\pi$. However, a better [3] overall match occurs for $C = 1.702^{-1}$, and this value was used in the simulations in Fig. 2b,d-e.

The generative model describes temporally extended outcome as continuous time series, but for the sake of practicality, we operate the model in the discrete domain in which perceived values are sampled and held constant throughout the sampling interval that is the duration of each stimulus. The unit of $\tau$ is seconds in the continuous domain, and in the discrete implementation we can characterize the decay in discrete units of stimulus onset asynchrony (*soa*) in terms of its half-life:

$$\tau_{0.5} = \frac{-\log(0.5)}{soa}\tau$$

In the discrete implementation, incentive value can be expressed in its recursive form: $y_n = (1 - a)y_{n-1} + x_n$, where $a = 1 - \exp(-w)$ as illustrated in Fig. 2a.

For two options of equal contents presented so that the temporal profile for one option is increasing and decreasing for the other option, as in Fig. 2b, preference for positive contrasts causes differential discount on the perceived values of the competing options. This mechanism leads to evidence in favour of the increasing profile, under the parameterization of $\tau$, $B$ and $\beta$. Notice that optimal accumulation of evidence is characterized by $\tau = \infty$ leading to $\epsilon_{\mathrm{A}} = \log(\int x_{\mathrm{A}} dt) - \log(\int x_{\mathrm{B}} dt)$. The parameters and variables of the model are summarized in Table S1.

*Alternative mechanisms*

Because the bounded dynamic range of neural encoding makes linear integration of the perceived values computationally intractable, especially for episodes of unknown duration, an observer may use numerosity or symbolic representations of reward value to construct summary evaluations for temporally extended outcomes. However, even for symbolically represented reward magnitudes, summary evaluation is facilitated by relative encoding [4]. Alternatively, the observer might adopt different valuation strategies for increasing and decreasing sequences. It is conceivable that expectations about upcoming events might affect some observers in ways that lead to differential valuation depending on expectation. However, such a strategy would require the observer to decide which strategy to follow before the experience begins. By contrast, leaky integration of the perceived values implement accumulation of evidence in the same way for all experiences. Thus, contrast-guided evaluation as described above is a biologically plausible and parsimonious mechanism for constructing summary evaluations for temporally extended outcomes.

**Simulations**

In order to test the ability of the proposed framework to characterize violation of dominance for temporally extended outcomes, the generative model was tested in several parametric implementations. Competing options with identical contents were used to simulate the mechanism in Fig. 2a. Each option had 19 elements; arranged along a decreasing or increasing profile as shown in Fig. 2b. The function $x = (E/100)^{\kappa}$ implements perceived value and the exponential filter $H = (z + 1/\tau)^{-1}$ implements accumulation of incentive value. Signal-to-noise ratio (SNR) was simulated by variance in the sampling noise $\varepsilon_{\mathrm{S}} \in N(0, \sigma_S^2)$, $\mathrm{SNR} = \mathrm{EV}(E)/\sigma_{\mathrm{S}}$, and sensitivity and biases of the decision maker were simulated by decision noise. Since it is the difference in the expected value of the decision noises that determines bias, and it is the combined variance that determines sensitivity, for the sake of simplicity we added all the decision noise to option A, $\varepsilon_{\mathrm{A}} \in N(\beta_A, \sigma_A^2)$, leaving $\varepsilon_{\mathrm{B}} = 0$.

*Simulation 1*

We first investigated the interaction of value function and decay in the accumulation of evidence. The decay parameter $\tau$ discounts perceived value in the accumulation of incentive value and the value function parameter $\kappa$ determines the absolute amount of this discount. We

simulated the relative discount on incentive value based on the expected value of evidence (*i.e.* no noise) over the ranges $0.34 \leq \kappa \leq 1$ and $1 \leq \tau \leq 21$ (Fig. S2, Fig. 2c).

*Simulation 2*

We then investigated the mutual representation of SNR, bias and sensitivity of the decision maker for choice data produced by the model. Here we used a fixed value function $x = (E/100)^{0.67}$ and exponential filter $H = (z + 1/21s)^{-1}$, and varied the specification of the decision and sampling noises. We used three decision noises with expected values 0, 1 and –1 (simulating bias) and standard deviations of 0.25. 0.75 and 0.5 (simulating sensitivity). The bias values are arbitrary and mainly serve to separate visually the psychometric functions in Fig. 2d. Three values of sampling noise variance $\sigma_S^2$ were calculated to simulate signal-to-noise ratios, SNR $\in \{0, 4, 8\text{dB}\}$. On each simulated trial, one value of decision noise was drawn for $\varepsilon_A$ and 38 values of sampling noise were drawn for $\varepsilon_S$ and added to the state elements of the two options.

*Simulation 3*

We finally simulated the interaction of SNR and decay in the accumulation of evidence. To investigate the effect of state sampling noise, three values of variance $\sigma_S^2$ were calculated to simulate signal-to-noise ratios, SNR $\in \{-4, 0, 4\text{dB}\}$. For each SNR, we simulated preference for the increasing profile for four values of decay, $\tau \in \{1, 3, 8, 21\text{s}\}$ (Fig. 2e).

**Procedures**

*Pre-experimental evaluation*

We measured the relationship between the physical size and perceived value of the virtual coins. The coins were presented to the subjects in a 3D projection to simulate the variation in volume. The state variable is coin volume, and objective value would be proportional to volume insofar as the specific value of a precious coin is given in prize units per mass unit (*e.g.* £/gr). This means that a coin scaled in diameter by a factor $m$ differs in volume by $m^3$ compared to an unscaled coin. If perceived value would correspond to objective value there would be a linear relationship between simulated volume and subjective evaluations ($\kappa = 1$). The perceived value of the virtual coins was determined by a Becker-DeGroot-Marschak (BDM) evaluation task.

The participants were given a 5£ budget, and they were instructed to place bids on each of 120 coins according to how much they felt each coin was worth. The coins varied in simulated volume from 1% to 100% of a reference coin that was shown on the screen before the bidding started. A randomly chosen subset of the bids were drawn to exchange the £5 budget to approximately 30 virtual coins of varying size and value according to a second-prize auction [5]. This set of coins would become the subject's endowment in the main experiment that followed. Before the bidding round, the reference coin of nominal value 100 pence (£1) was shown on the screen. A training round of 20 coins preceded the actual bidding round. Each coin was visible for 350ms following a response window showing a black screen with the text "Place a bid for the coin" for up to 5 seconds.

After the bidding round the participants watched a computer animation of the auction, in which one coin at a time was drawn from the total pool and the associated bid placed by the participant was shown against the computer's bid. For each coin drawn in this way, the computer placed a random bid drawn from a rectangular distribution without taking into account the specification of the coin at stake or the participant's bid. If the computer's bid was higher, the coin at stake was discarded and a new coin was drawn from the pool of bids without replacement. If the participant's bid was higher, they would buy the coin and they would pay the amount bid by the computer. This amount was then taken from their budget, and this procedure would continue until the budget was spent. In this way approximately 30 bids were realized to ensure the participants an initial endowment of virtual coins. This endowment had an expected value of £10 because the coins were obtained in a second prize action [5] consistent with the BDM method [6]. In the subsequent experiments the value of the endowment was adjusted according to the participants' performance in the task, and payment was made on the basis of the final adjusted value of the endowment at the end of the experiment.

*Experiment 1*

Experiment 1 was designed to test if participants were sensitive to the chronological configuration of perceived outcomes. We used as option alternatives a set of arbitrary temporal profiles and slight modifications to these profiles. Experiment 1a used the temporal profiles shown in Fig. 3a (top). They included 9 pairs of sequences of up to 8 coins, and each condition was repeated 30 times. In three conditions the last coin was omitted, so 7 coins

competed with 8 coins. In one case (Fig. 3a, top left), the contents were identical; only the order in which the coins were presented differed. Experiment 1b used the temporal profiles shown in Fig. 3a (bottom). They included 9 pairs of sequences of 10 coins, and each condition was repeated 20 times. In one case (Fig. 3a, bottom left), the contents were identical; only the order in which the coins were presented differed.

The CSs were randomized and balanced as follows: each CS pair was used twice in two different subjects to indicate either option of one condition. For example, $CS_1$ would predict $US_A$ and $CS_2$ would predict $US_B$ to one subject, while to another subject $CS_1$ would predict $US_B$ and $CS_2$ would predict $US_A$. $CS_1$ and $CS_2$ were not used together otherwise, but other subjects saw $CS_1$ and $CS_2$ in other combinations ($CS_1$ vs $CS_3$, $CS_2$ vs $CS_4$ *etc.*) in other conditions excluding $US_A$ and $US_B$.

*Experiment 2*

Experiment 2 was designed to test if evaluation of sequence duration interacted with temporal contrasts. In this experiment long sequences were offered against weakly dominated alternatives. Between trials, the long sequences varied in length between 15 and 19 coins. To each long sequence, a weakly dominated alternative was created by removing between 0 and 4 coins creating short sequences of 11 to 15 coins. Thus, the weakly dominating option is at least as good as the alternative [7]. A brute-force method was used to determine, which coins to remove without changing the average objective value of the remainder. We used two levels of steepness to examine the potential effect of valence abruptness. Two levels of variance were used to obscure the underlying average profile. Thus, the sequences did never proceed monotonically along the temporal profile; for the wider distribution of obfuscation noise (Fig. S1 top panels) the underlying average temporal profile was very unclear, while for the narrower distribution (Fig. S1 bottom panels) it was somewhat easier. In each version of the experiment, two control conditions used: 1) a decreasing and an increasing sequence with the same number of coins (11-19) and 2) a short (11-15 coins) flat sequence versus a long (15-19 coins) flat sequence (Fig. S1g-h). Since each trial used a different implementation of the average profile, the CS was only used to indicate the contents of an option within one trial.

Experiment 2a used 4 pairs of experimental profiles with conflicting features and two control conditions with univariate features. They included 6 pairs of sequences of between 11 and 19

coins, and each condition was repeated 16 times. The four main conditions included long (15-19 coins) decreasing sequences in competition with short (11-15 coins) increasing sequences (Fig. S1a-d). Eight subjects completed 16 repetitions of each condition drawn without replacement from the profiles illustrated in Fig. S1. Experiment 2b used 4 pairs of experimental profiles with the 2 surface features objective value and temporal contrast defined in a 2×2 factorial way, and the two control conditions with univariate surface features. They included 6 pairs of sequences of between 11 and 19 coins, and each condition was repeated 18 times. The four main conditions included long (15-19 coins) and short (11-15 coins) sequences (Fig. S1c-f). A decreasing sequence was in competition with an increasing sequence and either could be long while the other was short. Thirty-three subjects completed 18 repetitions of each condition drawn without replacement from the profiles illustrated in Fig. S1.

The temporal profiles were composed by scaling a sigmoid function to first generate a decreasing reference profile: $Q_n = I + (F - I)\left[1 + \exp\left(s(n - N/2)\right)\right]^{-1}$ with $N$ elements, initial scale $I$ and final scale $F$. The temporal profiles were then composed by calculating individual obfuscated magnitudes, $m_n = Q_n + O$ where the obfuscation $O \in N(0, c^2)$ was pseudo-randomized so that the average was the reference profile. Obfuscation was used to make less obvious the underlying average temporal profile. Increasing profiles were generated by inverting the sequence along the temporal dimension, and dominated alternatives to each profile were generated by removing elements from the longer profile. The values in Table S2 were used to generate the profiles shown in Fig. S1.

## Statistics and modelling

*Experiment 1*

To test the predictive leverage of simple proxies for decision value, we used multiple logistic regression on the preference data $P$:

$$\log\left(\frac{P}{1-P}\right) = B \times R$$

The regressor $R$ satisfies $E = R \times Q$, where $E = [\epsilon_{\mathrm{mean}}\ \epsilon_{\mathrm{proxy}}]$ is evidence calculated according to the proxies $\epsilon = \log\left(x_{\mathrm{A}}/x_{\mathrm{B}}\right)$, where $x_{\mathrm{I}}$ is perceived value (mean or proxy) of option $\mathrm{I} \in \{\mathrm{A}; \mathrm{B}\}$, and $Q$ is the modified Gram-Schmidt orthogonalization matrix [8]. We first fitted $B$ for the proxies initial and final values (Fig. 3b top). Next, to enable analysis of the

distribution of specific predictive leverage of coin position on preference for sequences of varying duration, we resampled all sequences to the maximum number of coins by linear interpolation. We then fitted $B$ to fixed-effects models in which $\epsilon_{\mathrm{proxy}}$ was characterized by the perceived values of each element in the resampled sequences (Fig. 3b bottom). Note that this approach cannot separate effects related to correlation between the proxies; it only removes the effect of mean value. Thus, each slope estimate indicates the predictive leverage of coin position for the residual.

*Experiment 2*

There were no effects of the steepness manipulations in Exp. 2a, so we restricted the subsequent analyses to the effects of biases and integration decay on dominated choice behaviour. One subject was excluded from the statistical analysis in Exp. 2b. For this participant, performance was at chance level throughout with no systematic preference for positive or negative contrasts or biases for any observable feature. This behaviour indicates that he did not seem to participate in the experiment in any meaningful way, and for that reason it seems unlikely that performance scores or response time (RT) parallel cognitive involvement in the task. The average RT in the experimental conditions in Exp. 2b was 1.11s (std 0.28s), with no statistically significant differences between conditions. By comparison with the experimental conditions, RT was slightly longer in the control condition with equal contents (average 1.18s, $t_{31}$ =3.0, $p$=0.0053) and similar in the flat control condition (average 1.14s, $t_{31}$ =1.1, $p$=0.29).

The average dominated choice score in Exp. 2a was 0.43 (std 0.11) compared with 0.29 (std 0.23) for the conflict conditions in Exp. 2b ($t_{38}$ =1.64, $p$=0.11), while the average score for the increasing option in the control condition with equal contents in Exp. 2a was 0.78 (std 0.16) compared with 0.60 (std 0.19) in Exp. 2b ($t_{38}$ =2.44, $p$=0.02). Although it seemed that the participants in Exp. 2a were more impressed by the temporal profiles than in Exp. 2b, the difference in dominated choice score was not statistically significant. For the flat control conditions, the average dominated choice score was 0.21 (std 0.19) in Exp. 2a and 0.20 (std 0.19) in Exp. 2b ($t_{38}$ =0.15, $p$=0.88).

We used a combination of nonlinear optimization algorithms implemented in MATLAB to estimate the parameters to each participant's full data set over the trials of all conditions.

First, the parameters of the individual value function were estimated using unconstrained 2-norm minimization of the state error in the pre-experimental evaluation. Using these data, we analyzed the underlying noise in state estimate. Then, the perceived values of each sequence were calculated according to individual value functions and evidence was derived according to each model. The parameters of the generative model outlined above were fitted to the choice data to investigate effects of biases and decay on revealed preference for the coin sequences. We examined the involvement of bias for the first option ($\beta_1$), bias for positive contrast ($\beta_+$) and decay parameter ($\tau$) using six separate models implementing decay and bias in a 2×3 factorial way [decay / no decay ($\tau$) × no bias / primacy ($\beta_1$) / positive contrasts ($\beta_+$)]. All models included the inverse temperature ($B$) of the stochastic process, so in total the models had 1 – 3 free parameters ($\tau$, $\beta$, $B$). For the sake of consistency all trials were included in all model fits. Because of the obfuscation noise, the flat control sequences had slight random slopes. We estimated the average slope in each trial and characterized the control trials according to the experienced slopes. We analysed Exp. 2a separately from Exp. 2b as the two versions of the experiment contained different sets of conditions with a higher prevalence of conflict trials in Exp. 2a than in Exp. 2b.

For each subject, the six models were fitted (Fig. S4) and the involvement of the parameters in explaining choice behaviour was examined by testing the fitness of the models with Akaike and Bayesian Information Criteria. They both evaluate the cost of fit but they penalize the number of free parameters in different ways. For AIC, the null model (no decay, no bias) was the best model in 5 participants. The most successful model according to AIC was the bias-free leaky integrator ($N = 13$) followed by positive-contrast bias ($N = 8$). For BIC the most successful model was the null model ($N = 14$) followed by the bias-free leaky integrator ($N = 12$) (Fig. S5). Since the null model can only account for variance in non-dominated choices, the question of which mechanism is best should pertain to the ability of the experimental models to account for dominated choices. Therefore, we may compare model fitness between the experimental models that are mechanistic candidates to capture variance related to violation of dominance. Within both information criteria, leaky integration and positive-contrast bias are the most successful experimental models. This result means that leaky integration of evidence and positive-contrast bias are the most likely individual strategies leading to violation of dominance. At the group level, group AIC and group BIC scores can be obtained by summing the scores across subject [9]. When the models are

compared at the group level, the best model fitness was obtained for the bias-free leaky integrator (Table S4).

Additional Analyses

Separate models were implemented to analyse decision bias for the first option and bias for positive contrasts. The predictors indicating option order and their respective temporal contrasts are independent, and the associated biases from the primary analyses were uncorrelated ($\rho^2 = 0.0052$, $p = 0.699$). Therefore, we tested a dual-bias model in which parameter estimates were obtained for primacy bias ($\beta_1$) and positive contrasts ($\beta_+$) by fitting the choice data ($P$) according to: $\text{logit}(P) = B(\epsilon + a_1\beta_1 + a_+\beta_+)$, where $\epsilon$ is evidence as defined earlier and $a_1$ and $a_+$ are indicator functions for option primacy and positive contrasts, respectively. There was high correlation between parameter estimates obtained with the dual-bias model and the separate models for both primacy ($\rho^2 = 0.87$, $p = 3.5\times10^{-14}$) and positive contrasts ($\rho^2 = 0.97$, $p = 8.5\times10^{-24}$). In no case did the dual-bias model outperform any of the other models according to any criterion.

In every trial, one of the options was weakly dominated by the alternative. Since the value function is non-linear, ambiguity in dominance relation was avoided by offering option alternatives composed of a coin sequence and a subset of that same sequence. Hence, in any trial the shorter option is dominated by the longer option regardless of the individual value function exponent $\kappa$. Thus, it is conceivable that it is not perceived value but rather state estimate that is discounted in the accumulation of evidence (although it is not immediately obvious how an internal representation of state would be divorced from its perceived value). To address this question, we re-fitted the parameters of the biasfree leaky integrator to the data using objective value in place of perceived value for all subjects. We analysed the difference in parameter estimates obtained with the simplified model that generally lead to a decrease in model fitness (Fig. S6).

**Response strategies**

We interviewed the participants at the end of the experiments and asked them how they had approached the task. Most participants reported a tendency to rely on a strategy whereby coins were classified in two or three bins (*e.g.* small/medium/large) and the number of large coins became the main determinant of choice. Some participants also reported that the coins

seemed to be presented along a temporal profile and they were mindful not to let it affect their judgment. No participant reported relying entirely on duration, option order or screen position. Thus, the participants appeared to operate analytically while trying to avoid contextual effects. In spite of these attitudes, a large number of choices revealed preference for the dominated option. This result suggests that the participants had no explicit access to the generative process underlying their choices.

## References


1. Stevens S.S. 1957 On the psychophysical law. *Psych Rev* **64**, 153-181.
2. Hodgkin A.L., Huxley A.F. 1952 A quantitative description of membrane current and its application to conduction and excitation in nerve. *J Physiol* **117**(4), 500-544.
3. Bowling S.R., Khasawneh M.T., Kaewkuekool S., Cho B.R. 2009 A logistic approximation to the cumulative normal distribution. *J Ind Eng Manag* **2**(1), 114-127. (doi:doi:10.3926/jiem.2009.v2n1.p114-127).
4. Livingstone M.S., Pettine W.W., Srihasam K., Moore B., Morocz I.A., Lee D. 2014 Symbol addition by monkeys provides evidence for normalized quantity coding. *Proc Natl Acad Sci U S A* **111**(18), 6822-6827. (doi:10.1073/pnas.1404208111).
5. Vickrey W. 1961 Counterspeculation, Auctions, and Competitive Sealed Tenders. *J Fin* **16**(1), 8-37. (doi:10.1111/j.1540-6261.1961.tb02789.x).
6. Becker G.M., DeGroot M.H., Marschak J. 1964 Measuring utility by a single-response sequential method. *Behav Sci* **9**(3), 226-232.
7. Nurmi H. 2006 *Models of Political Economy*, Taylor & Francis.
8. Golub G.H., Van Loan C.F. 1996 *Matrix Computations*, Johns Hopkins University Press.
9. Burnham K.P., Anderson D.R. 2002 *Model Selection and Multimodel Inference: A Practical Information-Theoretic Approach*. Berlin, Springer.

**Fig. S1 Profiles of temporally extended outcomes in experiment 2.**

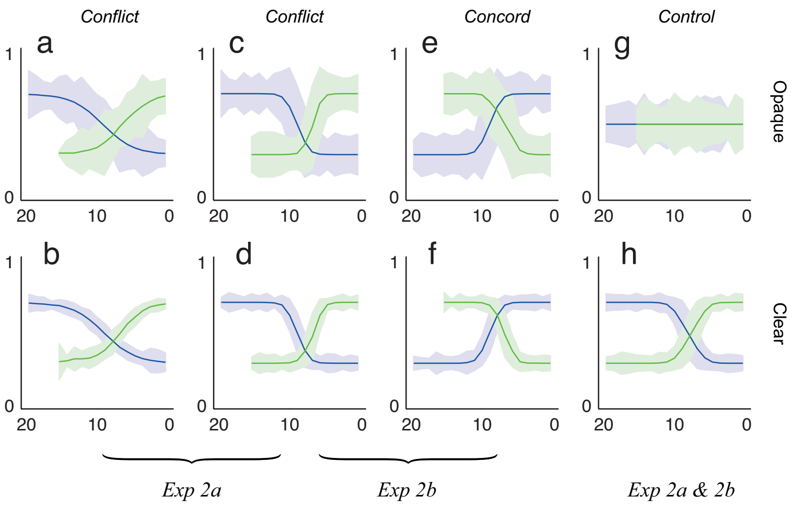


Temporal profiles plotted as a function of (remaining) sequence length. Each panel illustrates a pair of average temporal profiles in solid lines and the standard deviation of the distinct profiles in shaded area underneath. In the top row, coins were drawn from a wide distribution around the average profiles making the expected value of the temporal contrasts opaque to the observer. In the bottom row, the distribution was narrower making it clearer whether an option primarily consisted of positive or negative contrasts. (**a-d**) Pairs of profiles are in conflict with regard to their presumed appeal: the longer sequence is decreasing (containing a succession of negative contrasts) while the shorter sequence is increasing (positive contrasts); (**e-f**) pairs of profiles are in concord in this respect. (**g**) Control condition consisting of flat sequences (long vs short). (**h**) Control condition consisting of primarily positive- *vs* negative-contrast pairs with otherwise identical contents. In Exp. 2a, temporal profiles in conflict were tested for shallow and steep slopes and for clear and opaque temporal contrasts (panels a-d). Exp. 2b used a factorial design of positive *vs* negative contrasts and certain *vs* uncertain contrasts (panels c-f). The control conditions (panels g-h) were included in both versions of experiment 2.

**Fig. S2 Discount on incentive value over a range of decays and value function exponents.**

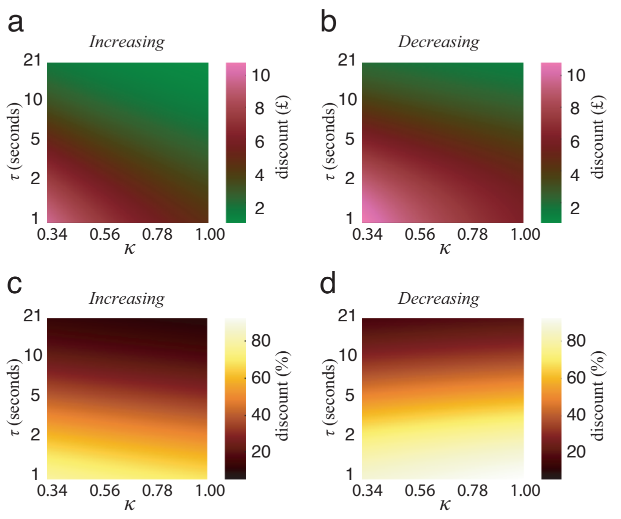


Discount landscapes of incentive value under parameterization of decay, $\tau \in [1; 21]$, and value function exponent, $\kappa \in [0.34; 1.00]$, showing that the deficit incurred in incentive value is largest for small values of $\tau$ (indicating a steep decay) and for small value of $\kappa$ (indicating a more concave value function). (**a**) Discount compared to linear integration for the increasing profile in Fig. 2b, and (**b**) discount compared to linear integration for the decreasing profile. (**c**) Discount for the increasing profile, and (**d**) for the decreasing profile relative to linear integration. The difference between the discount surfaces for increasing and decreasing profiles determines the deficit in incentive value incurred by the difference in the temporal profiles between the two options in Fig. 2b. Thus, Fig. 2c shows panel **d** above relative to panel **c** above.

**Fig. S3. Effects of opacity and evidence conflict on dominated choice**

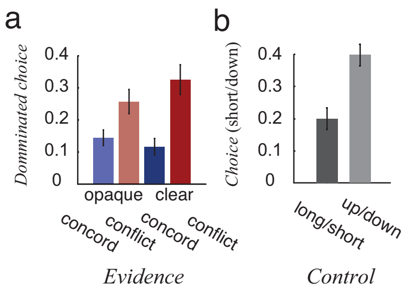


Effects of opacity and evidence conflict on dominated choice propensity (average +/- SEM) in Exp. 2b. (**a**) Repeated measures ANOVA: there was a main effect of evidence conflict ($F_{1,31}$ = 19.8, $p$ = 0.000104, $\eta_p^2$ = 0.39) and interaction with opacity ($F_{1,31}$ = 6.7, $p$ = 0.0145, $\eta_p^2$ = 0.18). (**b**) In the control conditions, dominated choice scores were significantly below chance; preference for the dominated shorter sequence was 0.2 ($t_{31}$ = 8.94, $p = 4.3\times10^{-10}$), and preference for the decreasing sequence was 0.3987 ($t_{31}$ = 2.97, $p$ = 0.0057).

**Fig. S4. Distribution of parameter estimates for all generative models and their relationships with dominated choice score**

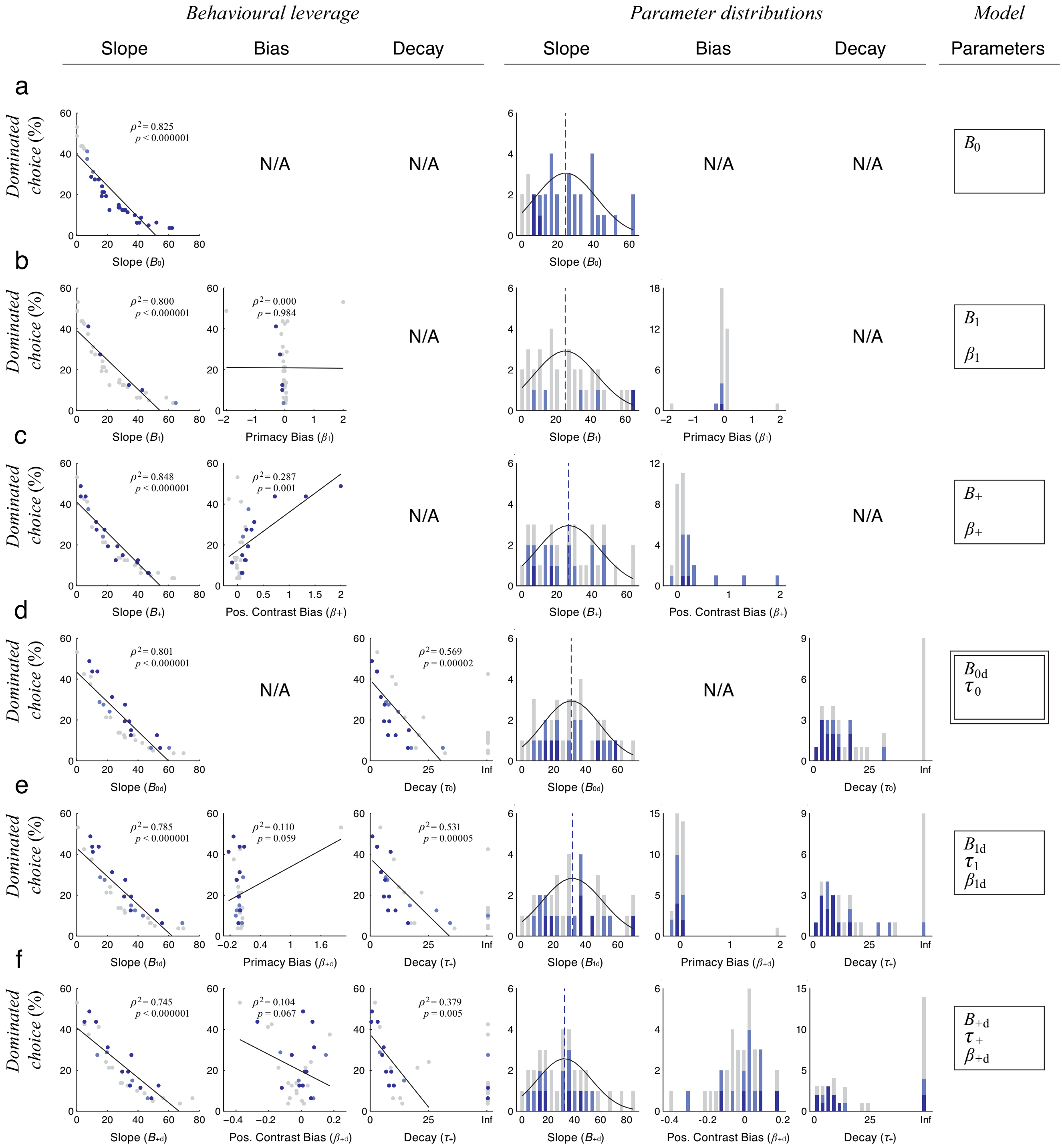


Distribution of parameter estimates for the six generative models and their relationships with dominated choice propensity in Exp. 2b. To the right are shown each model's parameters: psychometric slope (inverse temperature, $B$), bias ($\beta$) and decay ($\tau$). The relationship between

inverse temperature and dominated choice occurs because errors that are not caused by suboptimal integration are fewer in participants who are more sensitive to variation in evidence. This relationship is intrinsic to the structural effect of the parameter. By contrast, the relationship between decay and dominated choice indicates that the exponential filter is suitable for implementing suboptimal integration of evidence in participants who make many dominated choices. Cases in which the decay parameter converged to infinity are excluded in the calculation of the correlation coefficient ($\rho$). Histograms and scatter plots are colour-coded according to the $p$-value for the null model fit (a), or according to the $p$-value for the improvement in model fit over the null-model (b-f); grey: $p > 0.05$; light blue $p < 0.05$; dark blue $p < 0.01$. (**a**) Null model with only inverse temperature. (**b**) Model with bias for primacy and (**c**) for positive contrast. (**d**) Biasfree decay model with suboptimal integrator, and decay models with bias for (**e**) primacy and (**f**) positive contrast. The generative model allows for testing the effect of any kind of bias such as geometric or chromatic surface features of the CS. No systematic involvement was found for spatial orientation or any other kind of indexical information relating to the CS.

**Fig. S5. Model evaluation in Experiment 2b**

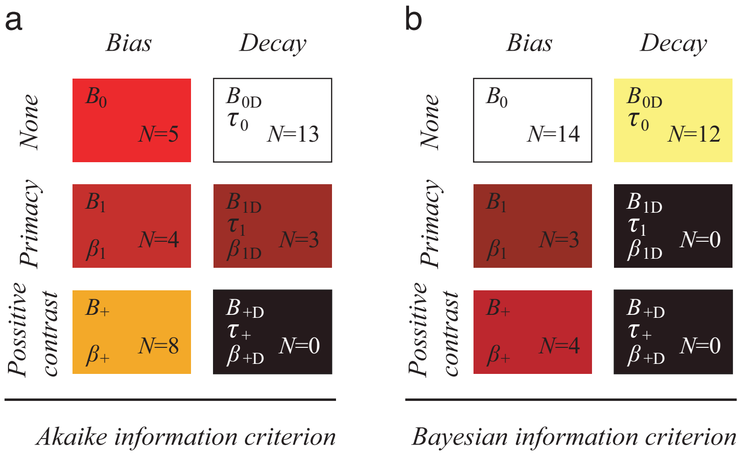


The six generative models indicate the involvement of inverse temperature ($B$), biases for primacy ($\beta_1$) and positive contrasts ($\beta_+$) and decay ($\tau$) in the observed choices. The boxes show model specification and the number of subjects ($N$) for whom it is the best model, tinted according to a hot colour map. (**a**) Model evaluation according to Akaike information criterion (AIC), and (**b**) Bayesian information criterion (BIC). BIC penalizes increase in parameter space more than AIC so none of the three-parameter models survive this criterion. The most successful two-parameter model is always the bias-free leaky integrator.

**Fig. S6. Simplifying the biasfree leaky integrator**

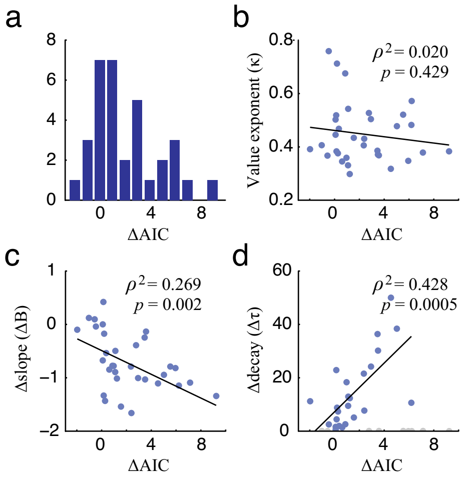


The cost in model fitness of removing individual value functions and the changes to parameter estimates for the simplified model. In some cases, removing variation in $\kappa$ results in a very similar model (*i.e.* little change in slope and decay) in which cases there is little or no increase in AIC for the simplified model. When the reduced model results in a worse fit (increase in AIC), the reduced model is characterized by a decrease in the slope of the psychometric function and an increase in the decay parameter for the exponential filter: (**a**) Distribution of the increase in AIC. (**b**) Relation between individual value function exponents ($\kappa$) and the increase in AIC caused by its removal. (**c**) Relation between the increase in AIC and the associated change in psychometric fit. A decrease in slope means that variation in evidence less precisely predicts observed choices. (**d**) Relation between the increase in AIC and the associated change in decay parameter estimate. An increase in decay reflects a reduced capacity of the exponential filter to effectively adjust the evidence underlying violation of dominance. When the decay was infinite in both models, the difference in decay was taken as zero (grey dots); these cases were excluded in the estimation of the trend line.

## Table S1. Model parameters

| *Symbol* | *Parameter* | *Relation* |
|---|---|---|
| $E$ | State | |
| $\varepsilon_{\mathrm{S}}$ | Sampling noise | $\varepsilon_{\mathrm{S}} \in N(\mu_{\mathrm{S}}, \sigma_{\mathrm{S}}^2)$ |
| $\hat{E}$ | State estimate | $\hat{E} = E + \varepsilon_{\mathrm{S}}$ |
| $x$ | Perceived value | $x = K\hat{E}^{\kappa}$ |
| $\kappa$ | Value function exponent | |
| $K$ | Value function scaling constant | |
| n | Sampling index for sequence of discrete state instances | |
| $x_{\mathrm{n}}$ | Sequence of perceived values | |
| $y_{\mathrm{n}}$ | Cumulative incentive value | $y_n = (1-a)y_{n-1} + x_n$ |
| $a$ | Recursive decay | |
| $\tau$ | Leaky integration decay | $\tau = -1/\log(1-a)$ |
| $t$ | Continuous time | |
| $x(t)$ | Episode of perceived value | |
| $y(t)$ | Cumulative incentive value | $dy/dt = x(t) - wy(t)$ |
| $w$ | Immediacy of contrast on marginal incentive | |
| $\tau$ | Leaky integration decay | $\tau = 1/w$ |
| $\rho_{\mathrm{A}}$ | Decision variable for option A relative to option B | $\rho_{\mathrm{A}} = y_{\mathrm{A}}/y_{\mathrm{B}}$ |
| $\epsilon_{\mathrm{A}}$ | Evidence in favour of option A | $\epsilon_{\mathrm{A}} = -\log(1/\rho_{\mathrm{A}})$ |
| $P_{\mathrm{A}}$ | Preference for option A | $\log(P_{\mathrm{A}}/(1-P_{\mathrm{A}})) = B(\epsilon_{\mathrm{A}} + \beta_{\mathrm{A}})$ |
| $B$ | Sensitivity to variation in evidence (inverse temperature) | $B^{-1} = C\sqrt{\sigma_A^2 + \sigma_B^2}$ |
| $C$ | Logistic scaling constant | $C = \sqrt{3}/\pi$ or $C = 1.702^{-1}$ [3] |
| $\beta_{\mathrm{A}}$ | Decision bias in favour of option A | $\beta_{\mathrm{A}} = \mu_{\mathrm{A}} - \mu_{\mathrm{B}}$ |
| $\varepsilon_{\mathrm{A}}$ | Decision noise option A | $\varepsilon_{\mathrm{A}} \in N(\mu_{\mathrm{A}}, \sigma_{\mathrm{A}}^2)$ |
| $\varepsilon_{\mathrm{B}}$ | Decision noise option B | $\varepsilon_{\mathrm{B}} \in N(\mu_{\mathrm{B}}, \sigma_{\mathrm{B}}^2)$ |

Model parameters and variables and their relation to other variables and parameters. The first section summarizes the perceived value function, the two middle sections summarize incentive value in the discrete and continuous domain, respectively, and the bottom section summarizes the decision model.

## Table S2. Specification of the utility profiles in Experiment 2

| Experiment | *N* | *I* | *F* | *s* | *c* | *M* |
|---|---|---|---|---|---|---|
| 2a | 19 | 0.3 | 0.7 | [0.5 2.0] | [0.05 0.15] | 16 |
| 2b | 19 | 0.3 | 0.7 | 2 | [0.05 0.15] | 18 |

Parametric specification of *M* different utility profiles in the second version of the monetary venture games (Experiment 2). *N* is the number of coins in the dominating reference sequence, and *I* and *F* are the initial and final scale values, respectively. The parameters *s* and *c* specify steepness of the temporal profile and variance in the obfuscation noise.

## Table S3. Individual data for the subjects in Experiment 2a

| | 1 | 2 | 3 | 4 | 5 | 6 | 7 | 8 |
|---|---|---|---|---|---|---|---|---|
| Score | 0.63 | 0.63 | 0.46 | 0.55 | 0.75 | 0.45 | 0.66 | 0.46 |
| $\kappa$ | 0.61 | 0.40 | 0.44 | 0.46 | 0.69 | 0.46 | 0.59 | 0.41 |
| $\beta_1$ | -0.09 | 0.12 | 0.37 | 0.48 | 0.10 | -1.75 | 0.00 | -0.11 |
| $\beta_+$ | 0.19 * | 0.34 *** | 2.00 *** | 0.55 *** | 0.16 ** | 0.67 *** | 0.07 | 0.59 *** |
| $\tau$ | 23.1 ** | 13.9 *** | 4.4 ** | 11.1 *** | 37.7 ** | 8.8 *** | Inf | 8.5 *** |

Individual parameter estimates for the subjects in Experiment 2a indicate that bias for positive contrast ($\beta_+$) and decay ($\tau$) parameters compete for the variance underlying dominated choices, whereas little involvement remains for bias caused by the primacy effect ($\beta_1$). * $p$<0.05; ** $p$<0.01; *** $p$<0.001. Choice frequency for the dominating options (Score) and individual value function exponent ($\kappa$) are shown for each subject.

## Table S4. Group comparison in Experiment 2b

| | Bias | | Decay | |
|---|---|---|---|---|
| *Criterion* | *AIC* | *BIC* | *AIC* | *BIC* |
| None | 3164 | 3247 | 2991 | 3156 |
| Primacy | 3167 | 3332 | 2996 | 3243 |
| Positive contrasts | 2995 | 3160 | 3006 | 3254 |

Model comparison at the group level in Experiment 2b according to Akaike Information Criterion (AIC) and Bayesian Information Criterion (BIC). For both criteria, the leaky integrator offers the best model fitness.
